# Placental P-glycoprotein inhibition enhances susceptibility to Di-(2-ethylhexyl)-phthalate induced cardiac malformations in mice: A possibly promising target for congenital heart defects prevention

**DOI:** 10.1371/journal.pone.0214873

**Published:** 2019-05-14

**Authors:** Changqing Tang, Chunyan Luo, Yimin Hua, Kaiyu Zhou, Hongyu Duan, Fan Ma, Yi Zhang, Yifei Li, Dajian Qiu, Chuan Wang

**Affiliations:** 1 Department of Pediatric Cardiology, West China Second University Hospital, Sichuan University, Chengdu, Sichuan, China; 2 West China Medical School of Sichuan University, Chengdu, Sichuan, China; 3 Department of Radiology, West China Hospital, Sichuan University, Chengdu, Sichuan, China; 4 The Cardiac development and early intervention unit, West China Institute of Women and Children’s Health, West China Second University Hospital, Sichuan University, Chengdu, Sichuan, China; 5 Key Laboratory of Birth Defects and Related Diseases of Women and Children (Sichuan University), Ministry of Education Chengdu, Sichuan, China; 6 Key Laboratory of Development and Diseases of Women and Children of Sichuan Province, West China Second University Hospital, Sichuan University, Chengdu, Sichuan, China; University of Missouri Columbia, UNITED STATES

## Abstract

**Backgrounds:**

Reducing toxicants transplacental rates could contribute to the prevention of congenital heart defects (CHDs). Placental P-glycoprotein (P-gp) plays a vital role in fetal toxicants exposure and subsequently affects the risk of toxicants-induced birth defects. However, data on the role of placental P-gp in decreasing toxicants-induced cardiac anomalies is extremely limited. This study aimed to explore the protective role of placental P-gp in reducing the risk of Di-(2-ethylhexyl)-phthalate (DEHP) induced cardiac anomalies in mice.

**Methods:**

The C57BL mice were randomly divided into four groups: the vehicle group (corn oil, n = 10), 500mg/Kg DEHP group (n = 15), 3mg/Kg verapamil group (n = 10) and 500mg/Kg DEHP & 3mg/Kg verapamil group (n = 20). Pregnant dams in different group received respective intervention by gavage once daily from E6.5–14.5. Maternal weights were monitored every day and samples were collected at E15.5. HE staining was used to examine fetal cardiac malformations. Real-time quantitative PCR (RT-qPCR) and Western-Blot were applied to detect Nkx2.5/Gata4/Tbx5/Mef2c/Chf1 mRNA and protein expression, respectively. The mRNA expression of peroxisome proliferator-activated receptor γ (PPARγ) was also determined using RT-qPCR.

**Results:**

Co-administration of verapamil and DEHP significantly elevated fetal cardiac malformation rates, in comparison with the DEHP group, the verapamil group and the vehicle group. Different phenotypes of cardiac anomalies, including septal defects and ventricular myocardium noncompaction, were noted both in the DEHP group and the DEHP & verapamil group. The ventricular myocardium noncompaction appeared to be more severe in the DEHP & verapamil group. Fetal cardiac PPARγ mRNA expression was notably increased and Gata4/Mef2c/Chf1 expression was markedly decreased in the DEHP & verapamil group.

**Conclusion:**

Placental P-gp inhibition enhances susceptibility to DEHP induced cardiac malformations in mice.

## Introduction

Worldwide congenital heart defect (CHD) is one of the most common birth defects, occurring in 7 to 8 per 1000 live births in China. The origins of CHDs are closely related to fetal toxicants exposure [1–4]. Previously, our epidemiological survey have illustrated that maternal exposure to phthalates, the most commonly used plasticizer, could increase the risk of CHD [5]. Thereafter, our animal study further proved that maternal exposure to di-(2-ethylhexyl)-phthalate (DEHP), which accounts for 80% of phthalate production in China, could result in various types of fetal cardiac anomalies in mice [6]. Except for merely exploring the mechanism regarding adverse effect of phthalates/DEHP on fetal cardiac development, seeking specific targets for decreasing transplacental transfer rates of phthalates/DEHP could be a promising alternative for CHDs prevention.

Substantial evidences have proved that placenta expresses a range of transporters, which are capable of controlling the transplacental disposition of massive xenobiotics and thus play a vital role in fetal protection against maternal toxins [7–12]. Of the special interest is ATP binding cassette (ABC) transporter, particularly the most characterized and widely one known as P-glycoprotein (P-gp). In human, P-gp was encoded by *ABCB1* gene alone, whereas two closely located genes (*Abcb1a* and *Abcb1b*) independently encode the isoforms of this transporter in rodents [13, 14]. Specially localizing at maternal-facing apical membrane of the syncytiotrophoblast, placental P-gp has the capacity to actively extrude a wide range of toxicants back to the maternal circulation, thereby prevent potentially harmful compounds from entering fetal compartment [7, 8, 13, 15, 16]. Studies in *Abcb1* knockout mice have verified that P-gp deficiency could result in many-fold higher concentrations of substrates in fetal compartment and could enhance susceptibility to chemicals induced birth defects [15, 17, 18]. However, association between the interindividual variability in placental P-gp expression and fetal susceptibility to toxicants induced CHD has rarely been explored.

Encouragingly, in our recent study, we have discovered that 3435C>T polymorphism of *ABCB1* gene could affect the placental P-gp expression, and then influence the effect of phthalates on the risk of CHD [19]. In light of these findings, by targeting placental P-gp, it appears to be plausible to reduce the transplacental transfer rates of phthalates and their adverse effects on cardiac development, and subsequently to decrease the general incidence of phthalates-induced CHDs. However, whether placental P-gp inhibition could indeed enhance the risk of phthalates induced fetal cardiac anomalies still remains elusive and waits to be confirmed in vivo. DEHP, which has been found to cause cardiac malformations during embryogenesis in our previous study, has been proved to be a substrate of P-gp [20]. Therefore, on the basis of previous findings, this study was carried out to further verify the protective role of placental P-gp in reducing the risk of fetal cardiac anomalies induced by DEHP exposure in mice.

## Materials and methods

### Animals

All C57BL mice (8–10 weeks of age) used were purchased from Sichuan University Animal Institution. They were identically housed, maintained on a 12 h light/dark cycle and had access to rodent chow and water ad libitum. Pregnancy was defined after the presence of vaginal plug and designated as embryonic day [E] 0.5. All animal experiments were conducted in accordance with the National Institutes of Health Guide and with the approval of the Sichuan University Committee for the Care and Use of Laboratory Animals.

### Maternal DEHP exposure, placental P-glycoprotein inhibition via verapamil and sample collection

Our previous animal study has proved that pregnant dams received DEHP (250mg/Kg, 500mg/Kg, 1g/Kg) intervention by gavage once daily from embryonic day (E) 6.5 to E14.5 could result in fetal cardiac anomalies, with a dose-dependent manner. In the present study, the dosage of 500mg/Kg was chosen because it was found that in comparison with 250mg/Kg and 1g/Kg, maternal exposure to 500mg/Kg DEHP could not only lead to the occurrence of fetal cardiac developmental disorders, but also had a relatively lower effects on the mortality of both dams and fetus at the same time [6].

Verapamil, as one of the calcium channel blockers, has been widely documented as both a substrate and a special inhibitor of P-gp [21–23]. Numerous researches both in vitro and in vivo have proved that verapamil could increase transfer rate of P-gp substrates in various tissues, including the blood-brain barrier and blood-placental barrier [24–28]. Most importantly, it had been verified that inhibition of P-gp by verapamil could significantly increase cellular accumulation of DEHP [20]. Thereby, to reduce the efflux effect of placenta P-gp on DEHP across placenta, verapamil was used in this study and its dosage (3mg/Kg) was based on a previous animal study conducted by el-Ashmawy, I. M. et al [28], founding that verapamil (3mg/Kg by gavage) could dramatically enhance the susceptibility of fetal developmental disorders induced by ivermectin via P-gp inhibition. Additionally, before conducting the present study, digoxin was used as a probe for evaluation of P-gp efflux function, and we have proved that verapamil (3mg/Kg by gavage) could indeed suppress P-gp efflux activity and elevate digoxin transplacental rate in a pilot study.

DEHP (Sigma-Aldrich) and verapamil (Sigma-Aldrich) were diluted in corn oil (Sigma-Aldrich) and saline for treatment, respectively. A total of 55 pregnant mice were randomly divided into four groups: the vehicle group (corn oil) with 10 animals, 500mg/Kg DEHP group with 15 animals, 500mg/Kg DEHP & 3mg/Kg verapamil group with 20 animals and 3mg/Kg verapamil with 10 animals. Pregnant dams in different groups received respective interventions by gavage once daily from E6.5-E14.5. Dosages were adjusted daily for body weight changes. E6.5-E14.5 was chosen as the treatment window because this period is of great importance for fetal cardiac development in mice. Maternal weights were monitored everyday and samples were collected at E15.5 after the dams were euthanized with carbon dioxide (CO2) followed by cervical dislocation. Uterine contents were examined to determine the numbers of live fetuses, dead fetuses and abortion fetuses. The thoracic cavities of all live fetuses were opened rapidly. Then the whole heart without surrounding tissues were separated, taken out from all the pups and cleaned in PBS gently, with 1/3 fixed in 4% formalin for hematoxylin and eosin (HE) staining and 2/3 immediately frozen in liquid nitrogen for protein and mRNA extraction.

### Fetal heart tissue hematoxylin and eosin staining

Fetal heart tissues were fixed in 4% formalin in PBS and subsequently treated for the histological study by dehydration (increasing alcohol concentrations, from 60% to absolute alcohol), mounting in xylene and immersion in paraffin. Then, the paraffin blocks were cut into 4 mm sections for hematoxylin and eosin staining. The slides were viewed under an Olympus SZX12 inverted microscope and the images were captured under the Olympus U‐CMAD3 camera.

### RNA extraction and cDNA synthesis

Total RNA was extracted from 50 to 100mg of the frozen heart tissues by 1ml TRIzol (Invitrogen, Life technologies, Carlsbad, CA) and subjected to quantitative and qualitative measurements using a spectrophotometer (GeneGuant 100, GE healthcare). 1μg of total extracted RNA was reverse transcribed to cDNA via Prime Script RT reagent kit with gDNA eraser (DRR047, Takara, Dalian, China) according to the manufacturer’s instructions.

### Real-time quantitative polymerase chain reaction (qRT-PCR)

qRT-PCR was performed with cDNA templates and SsoFast EvaGreen Supermixture (Bio-Rad Laboratories, Hercules, CA). Briefly, sequences were amplified using 5μl reaction mixture, 0.3μl forward primer (10 pmolL^-1^), 0.3μl reverse primer (10 pmolL^-1^), 3.4μl nuclease-free H2O and 1μl cDNA in a total volume of 10μl. PCR conditions were 39 cycles of 30s at 95°C, 30s at 60/60/56/56/59/59°C for *peroxisome proliferator-activated receptor γ (PPARγ)/Nkx2*.*5/Gata4/Tbx5/Mef2c/Chf1*, and 1min at 72°C, preceded by an initial denaturation of 3min at 95°C, and followed by a continuous melting curve from 65°C to 95°C. A validation experiment has been undertaken in which equal quantities of mouse heart cDNA were used. Similar amplification efficiencies and CT values of *Gapdh* were obtained for each group. The stability of *Gapdh* expression among different groups verified the application of this gene for an appropriate endogenous control for normalization. Moreover, we have confirmed that the amplifications efficiencies of all genes involved in our study (*PPARγ/Nkx2*.*5/Gata4/Tbx5/ Mef2c/Chf1*) were consistent across a range of template concentrations, all the slope of the amplification efficiency curves were more than 95% and efficiencies for the target genes (*PPARγ*/*Nkx2*.*5/Gata4/Tbx5/ Mef2c/Chf1*) and the endogenous control gene (Gapdh) were approximately equal. All samples were amplified in triplicates. The expression levels of *PPARγ*/*Nkx2*.*5/Gata4/Tbx5/ Mef2c/Chf1* transcripts were presented by the mean of triple tests. The relative expression levels of *PPARγ*/*Nkx2*.*5/Gata4/Tbx5/Mef2c/Chf1* mRNA were normalized to expression of *Gapdh* using 2^-△△Ct^ methods. The primer sequences used were shown in Table 1.

**Table 1:** Primer sequences used for real-time quantitative PCR.

Name of genes	Forward (5’-3’)	Reverse (5’-3’)
*PPARγ*	ATCAAGAAGACGGAGACAGACA	TGGAAGAAGGGAAATGTTGG
Nkx2.5	CACCCACGCCTTTCTCAGTC	CCATCCGTCTCGGCTTTGT
Gata4	CTGTCATCTCACTATGGGCA	CCAAGTCCGAGCAGGAATTT
Tbx5	CAAACTCACCAACAACCACC	GCCAGAGACACCATTCTCAC
Mef2c	TAATGGATGAGCGTAACAGACAGG	ATCAGACCGCCTGTGTTACC
Chf1	GACAACTACCTCTCAGATTATGGC	TAGCCACTTCTGTCAAGCACTC
Gapdh	CCCATCACCATCTTCCAGGAG	GTTGTCATGGATGACCTTGGC

PPARγ: peroxisome proliferator-activated receptor γ

### Western blot analysis

100mg fetal heart tissues were homogenized in buffer including 1ml RIPA (P0013B, Beyotime, China) and 10μl complete protease inhibitor cocktail (P8340, Sigma-Aldrich). The homogenate was then centrifuged at 12000g for 5min at 4°C. Protein concentration was determined using enhanced BCA protein assay kit (P0010S, Beyotime, China) in accordance to the manufacturer’s protocol. Total protein (50μg/lane) was separated on 10% SDS-polyacrylamide gel and transferred to polyvinylidene fluoride (PVDF) membranes (Millipore, Bedford, MA). The membranes were blocked in 5% BSA in Phosphate-buffered saline containing 0.1% Tween-20 (PBST). Thereafter, blocked membranes were incubated with overnight at 4°C with monoclonal primary antibody for Nkx2.5 (13921-1-AP, proteintech, 1:500)/ Gata4 (19530-1-AP, proteintech, 1:200)/ Tbx5 (13178-1-AP, proteintech, 1:200)/ Mef2c (10056-1-AP, proteintech, 1:200)/ Chf1 (10597-1-AP, proteintech, 1:500) and Gapdh (ab9484, Abcam, 1:7500). Following extensive washing with PBST, these membranes were incubated with a 1:10000 dilution of horseradish peroxidase-conjugated goat anti-rabbit immunoglobulin G (IgG) secondary antibodies (No.107724, ZSGB-BIO, China). After extensive washing with PBST, protein-antibody complexes were visualized by the enhanced chemiluminescene detection system. The relative optical density of the bands was measured using software Gelpro32 and was standardized against the Gapdh. We have validated the specificity of antibodies (Nkx2.5/Gata4/Tbx5/Mef2c/Chf1) through pre-incubation with peptide epitope and prevention of binding at 37kDa/45kDa/58kDa/63kDa/ 36kDa, respectively.

### Statistical analysis

Quantitative data are presented as Means ± SEM, while qualitative data are expressed as n%. All analyses are conducted with SPSS18.0 version. Shapiro-Wilk test and homogeneity test of variance were used to confirm that quantitative data from different groups come from a normal distribution and meet the homogeneity of variance. Differences of quantitative data among the different groups were determined by one-way ANOVA followed by a SNK multiple comparisons. Chi-square test was used to compare proportions and Fisher test was used if not matched. A 2-tailed p value < 0.05 was selected as the level of significance.

## Results

**Fig 1:**
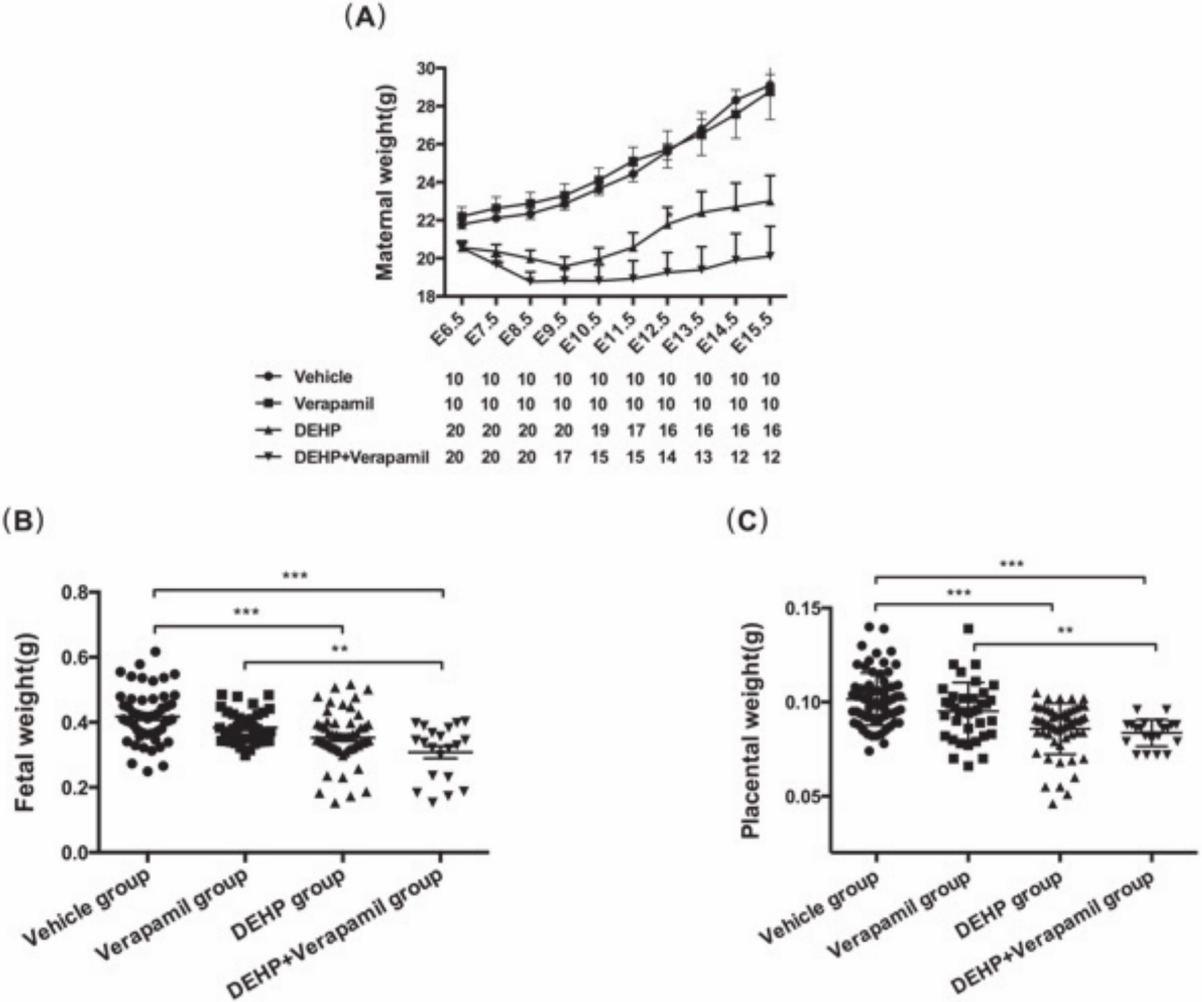
Effect of maternal Di-(2-ethylhexyl)-phthalate (DEHP) and verapamil exposure from E6.5 to E14.5 on maternal bodyweights (A), fetal weights (B) and placental weights (C). Differences among different groups were determined by ANOVA followed by a Student–Newman–Keuls multiple comparisons. N = 10, 15, 20 and 10 for vehicle, 500mg/Kg DEHP, 500mg/Kg DEHP & 3mg/Kg verapamil and 3mg/Kg verapamil group, respectively. Data were expressed as Means±SEM. *P<0.05, **P<0.01, ***P<0.001 in comparison with the verapamil group and the vehicle group.

As shown in Fig 1, the vehicle group and the verapamil group demonstrated similar maternal weight gain without significant difference. The dams both in the DEHP group and the DEHP & verapamil group showed a significantly declining trend of maternal bodyweight at each time point from E7.5 onwards compared with the verapamil group and the control group. By the time of sample collection at E15.5, maternal bodyweight in the DEHP & verapamil group was lower than that of the DEHP group. However, when the weight gain of the dam was adjusted with the number of the fetuses delivered by cesarean section, there were no significant differences in the weight gain between the DEHP group and the DEHP & verapamil group, indicating that the decreased body weight of pregnant dams was most likely caused by both a loss of the fetuses (the sums of dead fetuses, abortion fetuses, resorption fetuses and even the pre‐implantation loss), but not a toxic effect of DEHP on the dam itself. In addition, the fetus both in the DEHP group and the DEHP & verapamil group had general growth retardation; the bodyweights of live fetuses both in the two groups were significantly decreased at E15.5 in comparison with the vehicle group and the verapamil group. A significantly decreased placental weight was also observed in the DEHP group and in the DEHP & verapamil group.

**Fig 2:**
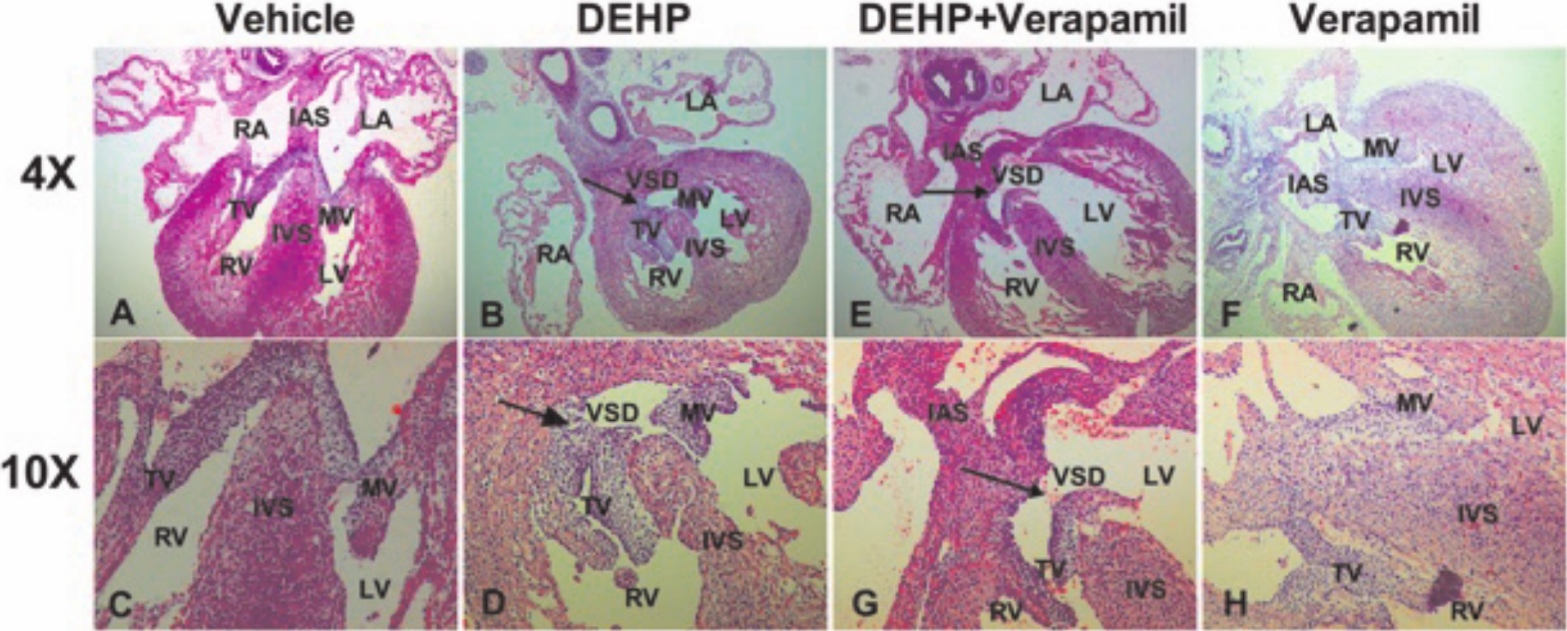
Fetal cardiac malformations induced by maternal Di-(2-ethylhexyl)-phthalate (DEHP) and verapamil exposure from E6.5 to E14.5. The phenotypes of fetal cardiac malformations included septal defects (B, D, E, G). The magnification used was 4×and 10×.

**Fig 3:**
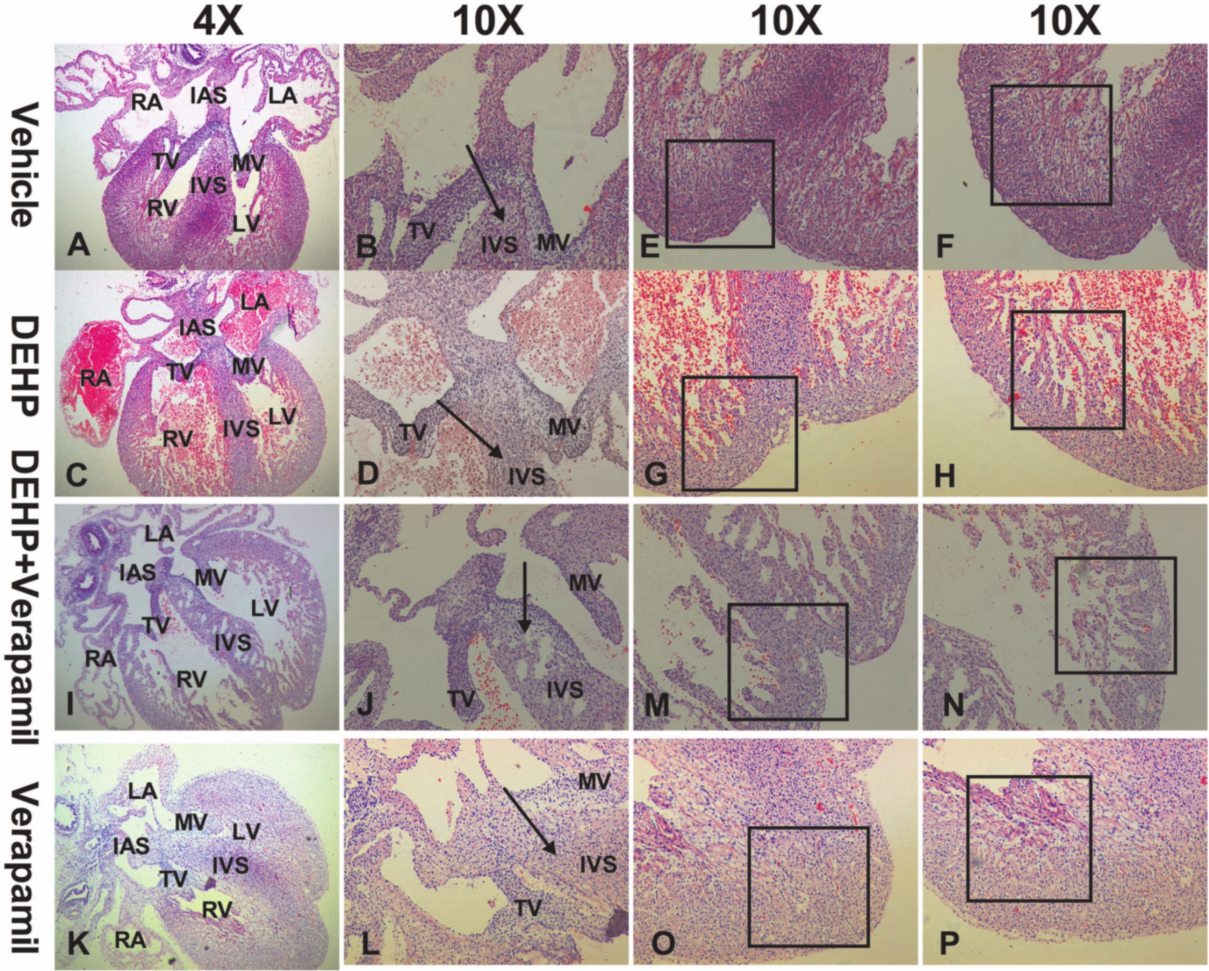
Fetal cardiac malformations induced by maternal Di-(2-ethylhexyl)-phthalate (DEHP) and verapamil exposure from E6.5 to E14.5. The phenotype of fetal cardiac malformations included ventricular myocardium noncompaction (G, H, M, N). The magnification used was 4×and 10×.

As Table 2 shown, there was significant difference in the incidence of cardiac malformation among different groups (P<0.001). After multiple comparisons using the Bonferroni P value adjustment, the results demonstrated that the incidence of cardiac malformation was significantly higher in DEHP group and in DEHP & verapamil group in comparison with the verapamil group and the vehicle group (35.0% vs 0.0%, P<0.001; 55.6% vs 0.0%, P<0.001), and the difference between the DEHP & verapamil group and the DEHP group was also apparent (55.6% vs 35.0%, P<0.001). However, there was no significant difference between the verapamil group and the vehicle group (0.0% vs 0.0%). The phenotypes of fetal cardiac abnormality both in DEHP group and in DEHP & verapamil group included septal defects (Fig 2B, 2D, 2E and 2G) and ventricular myocardium noncompaction (Fig 3G, 3H, 3M and 3N). Notably, it was found that the degree of myocardium noncompaction in the DEHP & verapamil group appeared to be more obvious than the DEHP group (Fig 3G, 3H, 3M and 3N).

**Table 2:** Comparison of fetal cardiac malformation rates among different groups.

Group	Vehicle	DEHP	DEHP & Verapamil	Verapamil	P value
No. of dams	10	15	20	10	-
No. of live fetus	78	60	27	72	-
No. of fetal cardiac HE staining	26	20	9	24	-
No. of VSD	0	1	2	0	-
No. of VMCNC	0	6	5	0	-
Fetal cardiac malformation rate (%)	0 (0.0)	7 (35.0)	5 (55.6)	0 (0.0)	<0.001^*^

DEHP: Di-(2-ethylhexyl)-phthalate; VSD: ventricular septal defect; VMCNC: ventricular myocardium noncompaction.

*Fisher test. Multiple comparisons using the Bonferroni P value adjustment were further conducted and the results demonstrated that the incidence of cardiac malformation was significantly higher in DEHP & verapamil group compared with the DEHP group, the verapamil group and the vehicle group (55.6% vs 35.0%; 55.6% vs 0.0%; 55.6% vs 0.0%; P<0.001), and the incidence of cardiac malformation in DEHP group also had a significant difference in comparison with the verapamil group and the vehicle group (35.0% vs 0.0%; 35.0% vs 0.0%; P<0.001), but there was no significant difference between the verapamil group and the vehicle group (0.0% vs 0.0%)

**Fig 4:**
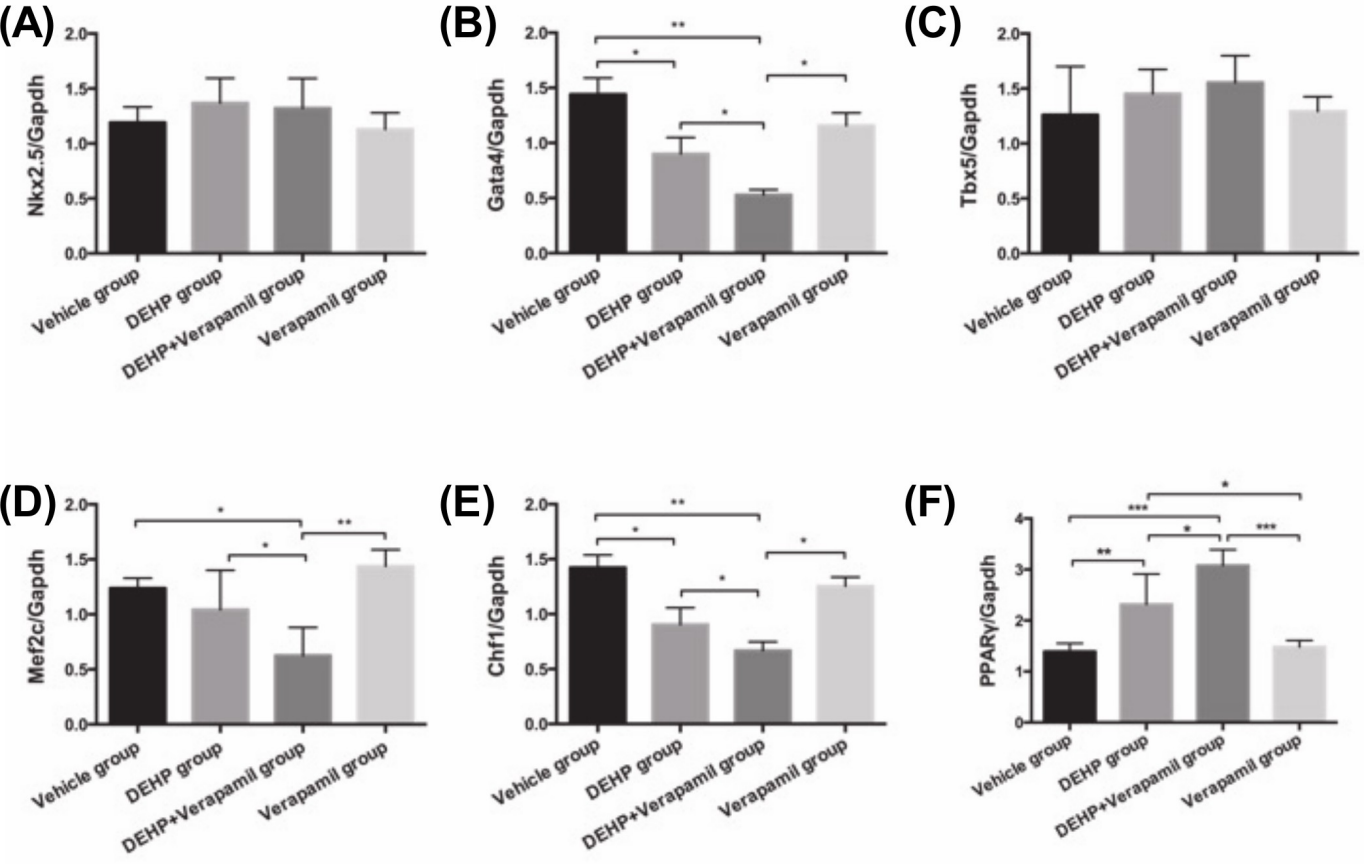
Effect of maternal Di-(2-ethylhexyl)-phthalate (DEHP) and verapamil exposure from E6.5 to E14.5 on fetal heart *PPARγ/Nkx2*.*5/Gata4/Tbx5/Mef2c/Chf1* mRNA expression at E15.5. Differences among different groups were determined by ANOVA followed by a Student–Newman–Keuls multiple comparisons. N = 10, 15, 20 and 10 for vehicle, 500mg/Kg DEHP, 500mg/Kg DEHP & 3mg/Kg verapamil and 3mg/Kg verapamil group, respectively. Data were expressed as Means±SEM. *P<0.05, **P<0.01, ***P<0.001 in comparison with the verapamil group and the vehicle group.

As shown in Fig 4, the mRNA expressions of both the *Nkx2*.*5* and *Tbx5* in fetal heart were not significantly different among all groups (all P>0.05). Fetal cardiac *Gata4/Mef2c/Chf1* mRNA expressions in the DEHP& verapamil group were notably decreased compared with the DEHP group, the verapamil group and the vehicle group (all P<0.05). The differences of both the *Gata4* and *Chf1* mRNA expressions in fetal heart between the DEHP group and the vehicle group were significant (all P<0.05), but without significances between the DEHP group and the verapamil group (all P>0.05). There was also no significant difference for the fetal cardiac *Mef2c* mRNA expression in the DEHP group compared with either the vehicle group or the verapamil group (P>0.05). Additionally, fetal cardiac *PPARγ* mRNA expression in the DEHP & verapamil group was evidently increased in comparison with the DEHP group (P<0.05), the verapamil group (P<0.001) and the vehicle group (P<0.001). *PPARγ* mRNA expression was also significantly higher in the DEHP group than that either in the vehicle group (P<0.01) or in the verapamil group (P<0.05). All mRNA expressions of fetal cardiac *Gata4/Mef2c/Chf1/PPARγ* were not comparable between the vehicle group and the verapamil group (all P>0.05).

**Fig 5:**
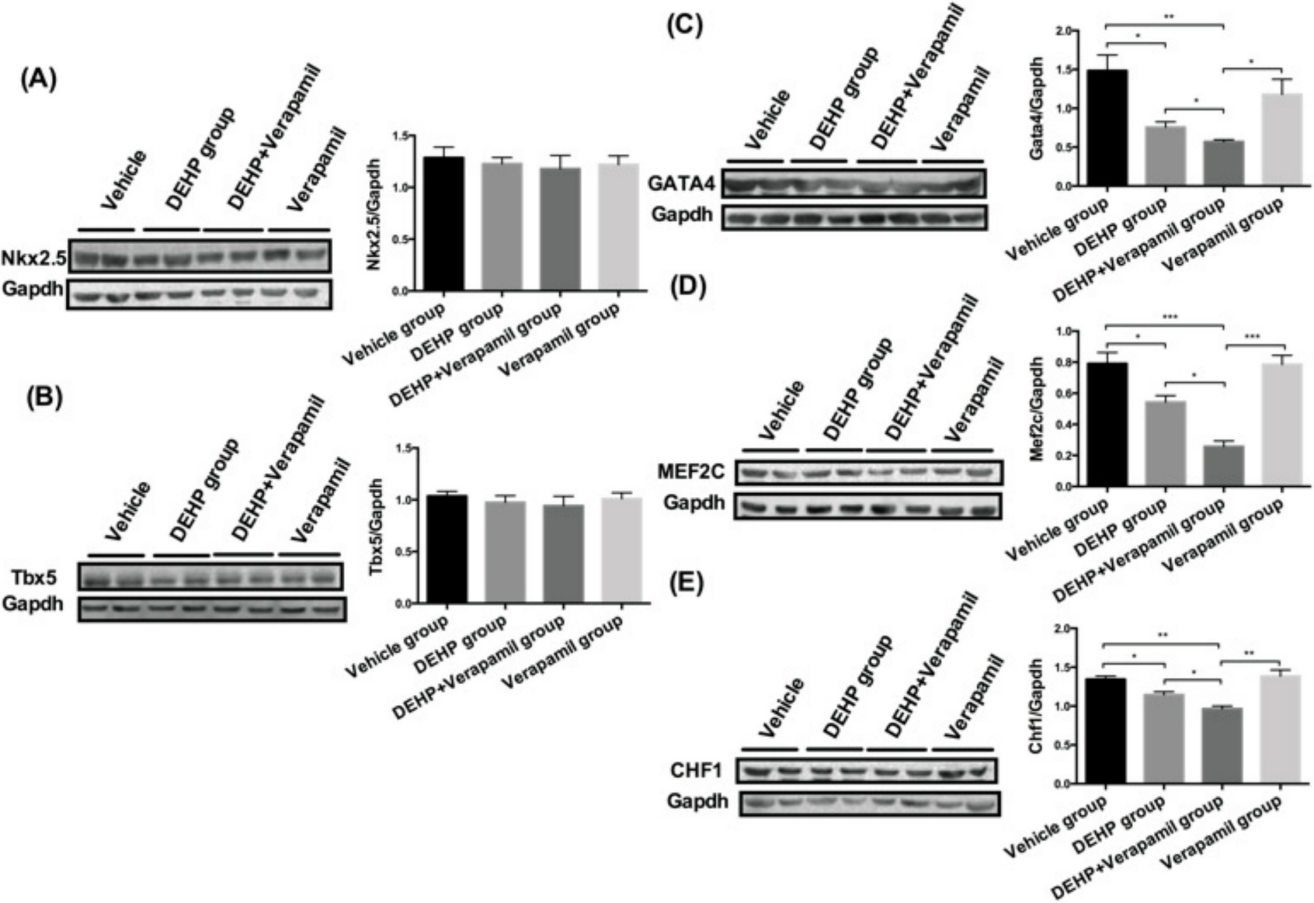
Effect of maternal Di-(2-ethylhexyl)-phthalate (DEHP) and verapamil exposure from E6.5 to E14.5 on fetal heart *Nkx2*.*5/Gata4/Tbx5/Mef2c/Chf1* protein expression at E15.5. Differences among different groups were determined by ANOVA followed by a Student–Newman–Keuls multiple comparisons. N = 10, 15, 20 and 10 for vehicle, 500mg/Kg DEHP, 500mg/Kg DEHP & 3mg/Kg verapamil and 3mg/Kg verapamil group, respectively. Data were expressed as Means±SEM. *P<0.05, **P<0.01, ***P<0.001 in comparison with the verapamil group and the vehicle group.

As shown in Fig 5, the results displayed that the protein expressions of *Nkx2*.*5/Tbx5* in fetal heart tissues were not obviously altered among different groups (all P>0.05). The fetal cardiac *Gata4/Mef2c/Chf1* proteins expressions in the DEHP & verapamil group were significantly down-regulated in comparison with the DEHP group, the verapamil group and the vehicle group (all P<0.05). The fetal cardiac *Gata4/Mef2c/Chf1* proteins expressions had also significant differences between the DEHP group and the vehicle group (all P<0.05), but without significances either between the DEHP group and the verapamil group (all P>0.05) or between the vehicle group and the verapamil group (all P>0.05).

## Discussion

Highly expressed early in fetus and placenta, P-gp appears to be one of the most characterized and abundant transporter in placenta [7–11, 13]. Several studies had displayed that placental P-gp had a protective role in fetal development by restricting the penetration of xenobiotics from maternal circulation into fetal compartment [9, 13, 29, 30]. As far back as 1998, an animal study performed by Lankas GR et al.[17]had found that fetuses deficient in *Abcb1* (-/-) were 100% susceptible to cleft palate resulting from exposure to avermectin, a known teratogenic substrate of P-gp, whereas the heterozygotes (-/+) littermates were less sensitive and the homozygous (+/+) fetuses with abundant P-gp were totally protected from the effects of teratogens. Afterwards, another study [15] revealed the similar results and they noticed that the mice strains with knockout *Abcb1* genes showed susceptibility to cleft palate following the administration of phenytoin to the pregnant dams. Among 48 fetuses with cleft palate, 36 had the heterozygous (*Abcb1a+/-*) genotype and 12 were homozygous for the knockout gene (*Abcb1a-/-*). Additionally, el-Ashmawy, I. M. et al., [28] observed that verapamil could dramatically enhance the susceptibility of fetal developmental disorders induced by ivermectin via P-gp inhibition. With regard to clinical studies, two researches [31, 32] have reported that 3435C>T polymorphism of *ABCB1* gene could affect the risk of toxicants-induced birth defects, which might be explained by the alteration of placental P-gp expression and efflux activity. Moreover, a recent epidemiological case-control survey [33] documented that several drug classes that are substrates for P-gp were shown to have a higher user rate in mothers of cases with specific anomalies. The use of this subset of drugs in combination with other P-gp substrates increased the risk for specific anomalies (OR 4.17, 95% CI 1.75–9.91), and the addition of inhibitors further increased the risk (OR 13.03, 95% CI 3.37–50.42). These findings discussed above provided strong evidences that placental P-gp play a vital role in fetal toxicants exposure and subsequently affect the risk of toxicants-induced birth defects. However, up to date, data on the role of placental P-gp in toxicants-induced cardiac developmental malformations is extremely limited.

On the basis of our previous epidemiological and animal studies revealing that 3435C>T polymorphism of *ABCB1* gene could influence the risk of CHDs when the mothers were exposed to phthalates, possibly through regulating placental P-gp expression, and maternal DEHP exposure could cause various types of cardiac anomalies in mice [6, 19], the present observational study was the first time and further confirmed the protect role of placental P-gp in DEHP induced fetal cardiac developmental disorders in vivo. We found that the incidence of fetal cardiac abnormalities in the DEHP & verapamil group was remarkably higher than that of the DEHP group, while the degree of myocardium noncompaction was more severe and apparent in the DEHP & verapamil group compared with the DEHP group. Additionally, the fetal cardiac PPARγ mRNA expression was significantly increased and both mRNA and protein levels of fetal cardiac Gata4/Mef2c/Chf1 expression were also markedly decreased when verapamil and DEHP were co-administrated. These results suggested that the inhibition of placental P-gp by verapamil, at least, partly enhance the susceptibility to DEHP induced fetal cardiac malformations. Currently, it was quite difficult to reduce the overall incidence of CHDs because almost all researches regarding CHD prevention merely focused on the effect and related mechanism of one or several toxicants on fetal cardiac development. Taken the findings in the present study, placental P-gp is most likely to become a promising target for CHD prevention. Elucidating the up-regulation mechanism of placental P-gp, seeking the specific targets for reducing the transplacental transfer rates of toxicants and their adverse effects on cardiac development, and subsequently forwarding CHD prevention front, might provide a brand-new insight for primary prevention of CHD in the profile of placenta. However, more studies are warranted to shed light on this issue.

Several limitations needed to be addressed. Firstly, verapamil was used to suppress placental P-gp efflux function in the present study, we could not rule out the possibility that verapamil could interact with some other placental transporters or metabolic enzymes, which perhaps in turn influence the transplacental rates of DEHP and its adverse effect on cardiac development. However, verapamil had been widely proved to be a relatively specific inhibitor of P-gp and was commonly used *in vitro* and *in vivo* studies for P-gp inhibition [21–28]. Additionally, DEHP was also a relatively specific substrate of P-gp, and to our knowledge, other transporters involving DEHP transfer has rarely been reported. Moreover, the subtypes of functional transporters and metabolic enzymes, which could be inhibited by verapamil and simultaneously transfer DEHP, might be limited. Most importantly, it has been proved previously that P-gp inhibition by verapamil could notably elevate cellular concentration of DEHP [20]. Therefore, the increased susceptibility of DEHP induced cardiac anomalies observed in the present study, was at least, partly owing to placental P-gp inhibition by verapamil. Secondly, since the HPLC/MS method for fetal-unit DEHP concentration determination was not validated, whether DEHP transplacental transfer rate indeed increased after co-administration of verapamil was undetermined. Nevertheless, additional effort had been made to make up this limitation. Before conducting the present study, we had conducted a preliminary study to verify that the efflux activity of placental P-gp was indeed inhibited after co-administration of verapamil, evidenced by increased transplacental rate of digoxin that is a pharmacological probe for placental P-gp function both evaluation. On the other hand, our previous animal study [6] had found that DEHP could increase fetal cardiac anomalies rates, decrease *GATA4/Mef2c/Chf1* expression and elevate *PPARγ* expression in fetal heart with a dose-dependent manner. In the present study, it was revealed that fetal cardiac anomalies rates were markedly increased and the expressions of *GATA4/Mef2c/Chf1* were notably decreased and the mRNA expression of *PPARγ* was also remarkably increased in DEHP & verapamil group compared with DEHP group. These findings strongly support that placental P-gp efflux function was indeed suppressed by verapamil and DEHP transplacental transfer rate increased. But, studies with placental *Abcb1* gene knockout mice and DEHP concentration measurement still need to be further carried out to strengthen the reliability of our findings. Lastly, we only explored P-gp inhibition on the risk of DEHP-induced cardiac malformations, other toxicants, such as sodium valproate, which could lead to cardiac anomalies and is also P-gp substrates, needed to be determined in the future.

Taken together, our group made a preliminary attempt and effort to explore the protective role of placental P-gp in toxicants-induced CHDs. The present observational vivo study, for the first time, provided a novel finding that placental P-gp inhibition, at least, partly increased susceptibility to DEHP induced cardiac malformations in mice. It was of great significance that placental P-gp might become a new target for CHDs primary prevention.
